# Thermal Carrying Capacity for a Thermally-Sensitive Species at the Warmest Edge of Its Range

**DOI:** 10.1371/journal.pone.0081354

**Published:** 2013-11-25

**Authors:** Daniel Ayllón, Graciela G. Nicola, Benigno Elvira, Irene Parra, Ana Almodóvar

**Affiliations:** 1 Department of Zoology and Physical Anthropology, Faculty of Biology, Complutense University of Madrid, Madrid, Spain; 2 Department of Environmental Sciences, University of Castilla-La Mancha, Toledo, Spain; University of Kent, United Kingdom

## Abstract

Anthropogenic environmental change is causing unprecedented rates of population extirpation and altering the setting of range limits for many species. Significant population declines may occur however before any reduction in range is observed. Determining and modelling the factors driving population size and trends is consequently critical to predict trajectories of change and future extinction risk. We tracked during 12 years 51 populations of a cold-water fish species (brown trout *Salmo trutta*) living along a temperature gradient at the warmest thermal edge of its range. We developed a carrying capacity model in which maximum population size is limited by physical habitat conditions and regulated through territoriality. We first tested whether population numbers were driven by carrying capacity dynamics and then targeted on establishing (1) the temperature thresholds beyond which population numbers switch from being physical habitat- to temperature-limited; and (2) the rate at which carrying capacity declines with temperature within limiting thermal ranges. Carrying capacity along with emergent density-dependent responses explained up to 76% of spatio-temporal density variability of juveniles and adults but only 50% of young-of-the-year's. By contrast, young-of-the-year trout were highly sensitive to thermal conditions, their performance declining with temperature at a higher rate than older life stages, and disruptions being triggered at lower temperature thresholds. Results suggest that limiting temperature effects were progressively stronger with increasing anthropogenic disturbance. There was however a critical threshold, matching the incipient thermal limit for survival, beyond which realized density was always below potential numbers irrespective of disturbance intensity. We additionally found a lower threshold, matching the thermal limit for feeding, beyond which even unaltered populations declined. We predict that most of our study populations may become extinct by 2100, depicting the gloomy fate of thermally-sensitive species occurring at thermal range margins under limited potential for adaptation and dispersal.

## Introduction

Natural and anthropogenic disturbances are impacting global ecological systems and causing elevated rates of population extirpation, so that there is increasing concern that the rate of environmental change may exceed the capacity of populations to persist and maintain their range [1]. A population's extinction risk, persistence time and duration of its final decline to extinction, as well as the probability of evolutionary rescue, strongly depend on initial numbers and population size variability [2]–[4]. In many systems, imminent extinction will be signalled early by a decreasing rate of recovery from small perturbations. This critical slowing down is typically characterized by an increase in variance or autocorrelation of fluctuations of the system as a tipping point is approached [5]–[6]. In highly stochastic systems, critical transitions will on the contrary happen far from local tipping points and an increasing variability will reflect the shift to a contrasting regime [7]. Improving wildlife's conservation and management requires therefore a deep comprehension of not only spatial patterns in local species abundance but also the way and rate a population's size changes through time - its trajectory. Since dynamics are driven by the interplay of density-dependent and density-independent aspects of the environment, determining how the strength of density dependence varies with environmental variance remains critical for predicting near-term population trajectories [8]–[9]; the heart of the matter is then, what limits and regulates the size of natural populations in a fluctuating world?

Theoretically, there is a limit to the maximum number of individuals that can be supported by a system over a period of time for a particular level of resources (i.e., the environment's carrying capacity); and most important, population growth must decrease as the population approaches that limit at a rate dependent on the functional form of density dependence operating on the system [10]. This latter notion has been factually the cornerstone of the management of wildlife populations subject to commercial exploitation (see [11]–[12]). In conservationist settings, the probability of extinction and the persistence time of a population are a function of the environment's carrying capacity and the amplitude of its fluctuations along time (e.g., [13]). Carrying capacity is however typically set as a static parameter in predictive population dynamics models notwithstanding the fact that levels of resources naturally change through time, and that these changes will be amplified by climate change in many regions. Mechanistic behavioural process-based models provide a useful alternative to simulate carrying capacity dynamics under changing conditions across multiple spatio-temporal scales, contexts and taxa (e.g., [14]–[16]). Yet most models do not account for social interactions even though the carrying capacity of an environment is greatly determined by how individuals compete over the available resources. This is especially relevant for territorial species because behavioural responses induced by aggressive interactions typically result in reduced exploitation of the limited resource, so that the population stability-enhancing effects of territoriality are paid-off by decreased carrying capacity [17].

In addition to the resources that set the carrying capacity, which are dynamically consumed and may be hence the object of competition, there are scenopoetic variables that are not dynamically affected by the presence of a species but may limit the species' final performance in the other way round. As such, temperature is a primary driver of species' distribution and numbers over the long-term, especially in ectotherm organisms, as their fundamental niche is physiologically bounded by their thermal niche space [18]. Within the temperature range in which survival occurs, there are a series of decreasing ranges for different functions (e.g., feeding, growth, reproduction) so that outside their limits population performance declines (e.g., [19]). Therefore, increasing temperatures may first constrain the carrying capacity of a system for a particular species to a lower thermal capacity and ultimately drive that organism outside its tolerance window.

Alterations in the realized thermal niche resulting from on-going anthropogenic global warming is in fact the underlying cause of the rapid range shifts [20], local and worldwide extinctions [21], and population declines [22] observed in species from a wide variety of taxa. Ominously, range sizes and population numbers of thermally-sensitive species are projected to keep on shrinking along warmer margins (latitudinal or altitudinal), with particularly deleterious impacts on peripheral populations living at the most extreme margins of the species' realized climatic niche (e.g., [23]–[25]). There is also increasing evidence that the amplitude and probability distribution of environmental variability is changing in response to anthropogenic impacts [9], with the intensification of weather and climate extremes linked to anthropogenic climate change at the far-end of this spectrum (see [26]). This trend can have a substantial influence on population extinction dynamics since increased environmental variability can alter a population's vital rates in several interrelated ways [9]. Understanding then the way and to what extent the internal dynamics of a system responds to temperature fluctuations over time is critical for predicting trajectories of change under future scenarios.

In this article, we address how the carrying capacity and the thermal capacity of the system act and interact to drive spatial patterns and temporal fluctuations in population abundance of thermally-sensitive species. For this purpose, we tracked during 12 years 51 populations of a cold-water fish species, brown trout *Salmo trutta*, living along a temperature gradient at the warmest thermal edge of its range. In this study, we develop a carrying capacity model in which maximum population size is limited by environmentally-driven physical habitat conditions and regulated through habitat selection and territorial behaviour. We test whether the spatial and temporal variations in the numbers of young-of-the-year, juvenile and adult brown trout (1) are explained by modelled carrying capacities, and (2) are disrupted by thermal conditions. If so, we target on establishing (3) the temperature range within which the thermal capacity of the system is lower than its carrying capacity, and (4) the rate at which carrying capacity declines with temperature within that limiting thermal range.

## Materials and Methods

### Study populations

The study area was situated in the Iberian peninsula between latitudes 42°29′ and 43°16′N and longitudes 0°43′ and 2°20′W. Brown trout population trajectory was analysed in 37 study sites located in 22 rivers from three major basins (Aragón, Arga and Ega river basins) belonging to the Ebro river basin, a Mediterranean drainage; 14 sites located in 12 Atlantic rivers from the Bay of Biscay drainage were additionally studied. Sampling sites corresponded to first- to fifth-order rivers and were located at an altitude ranging from 40 to 895 m above mean sea level (a.s.l.). Selected sites were chosen to (1) cover the existing variability of environmental (physical habitat, flow, water temperature) conditions, and (2) represent an anthropogenic multiple-stressor gradient within the study area. Location and environmental and physical features of sampling sites can be checked elsewhere (e.g., [27]–[29]). Brown trout is the dominant fish species throughout the area, and its populations only consist of stream-dwelling individuals.

Brown trout populations were sampled by electrofishing with multiple successive passes every summer from 1993 to 2004. Prior to sampling, each site was blocked upstream and downstream with nets. Trout were placed into holding boxes and were anaesthetised with tricaine methane-sulphonate (MS-222) to both facilitate their manipulation and minimize physiological stress. Individuals were measured (fork length, to the nearest mm) and weighed (to the nearest g), and scales were taken for age determination. Scales were removed from the area between posterior edge of dorsal fin and the lateral line, approximately two scale rows above the lateral line on the left side of the fish. Scales were removed by gently scraping against the grain of the scales with the blade of a clean scalpel or knife. After sampling routines, trout were placed into different holding boxes to recover, being immediately released back into the river after recovery. A grand total of 159,563 individuals were sampled during the study. Population density was used as a measure of population size. Fish density (trout ha^−1^) with variance was estimated separately for each sampling site by applying the maximum likelihood method [30] and the corresponding solution proposed by Seber [31] for three removals assuming constant-capture effort. Population estimates were carried out separately for each year class.

### Ethics statement

The described field study, including electrofishing and all sampling routines, was approved by the Wildlife Regional Service of Navarra (Department of Rural Development and the Environment and Local Administration of the Government of Navarra) accordingly to the current legislation (Ley Foral 2/1993). The study did not require any ethical approval from the corresponding Ethics Committee on Animal Experimentation (Ley Foral 2/1993, article 9; Real Decreto 1201/2005, articles 2–3). Fish surveys were carried out by experienced fisheries staff of the Wildlife Regional Service of Navarra and all sampling procedures complied with the Spanish and European Union legislation on animal welfare. The fish were handled with great care throughout this study to minimize any negative effects. This includes electrofishing and sampling routines such as weighing and length measuring and scale collection. After sampling routines, all fish were returned alive into the river. The study did not involve field sampling during the emergence or spawning critical periods, when trout are more susceptible to undergo potential physiological or behavioural disruptions. The described field studies did not involve endangered or protected species.

### Carrying capacity modelling

Carrying capacity dynamics was modelled following the rationale and methodology described in Ayllón et al. [32]. We define carrying capacity as the maximum density of fish a river can naturally support during the period of minimum available physical habitat. In our model, the quantity of suitable physical habitat available for fish of a given age is estimated as a function of stream discharge using physical habitat simulations, and the maximum number of fish that can be sustained is estimated as the area of suitable physical habitat divided by the expected individual territory area for the given aged cohort.

Dynamics of stream physical habitat was modelled by means of the Physical Habitat Simulation system (PHABSIM; [33]). PHABSIM simulations determine the potentially available physical habitat for an aquatic species and its life stages as a function of discharge by coupling a hydraulic model with a Habitat Suitability Model (HSM). The longitudinal distribution of habitat types within the stream is described through transects positioned perpendicular to the channel. Along each transect, measurements of physical habitat variables are made at regular intervals to describe their cross-sectional distributions. As a result, the study site is depicted as a mosaic of cells characterized by their area, structure (substrate and cover) and hydraulics (water depth and velocity), which are a function of discharge [34]. For this work, topographic, hydraulic and channel structure data needed to carry out the physical habitat simulations were collected at each study site during the summer of 2004 following survey methods extensively described for e.g. in Parra et al. [29]. Hydraulic conditions were simulated following procedures set out in Waddle [34]. Finally, the suitability of channel structure and simulated hydraulic conditions for fish is assessed by means of the HSM. In this study, we used the reach-type specific habitat selection curves developed for young-of-the-year (YOY, 0+), juvenile (1+) and adult (>1+) brown trout by Ayllón et al. [27]. Habitat selection represents habitat preference under the prevailing biotic and abiotic conditions in any particular system, so these curves can be seen as operational applications of the realized ecological niche. The curves that relates the Weighted Usable Area (WUA; m^2^ WUA ha^−1^, an index combining quality and quantity of available physical habitat) for each life stage with stream discharge are the final outputs of PHABSIM simulations.

Importantly, we modelled spatial segregation of cohorts to avoid an overestimation of available physical suitable habitat. Since habitat selection patterns overlap among life stages up to a certain degree (see [27]), there is a potential for intercohort competition in some areas of the stream. This results in PHABSIM cells where one life stage has a higher composite suitability index than other life stage, and other cells where the converse holds. We considered that younger life stages would not occupy the shared cells where they are dominated (have less favourable habitat conditions) by older ones, so that this WUA was not added to their total available physical habitat.

Discharge time series for the study period (1993–2004) were obtained at each site from the closest gauging stations. Then, summer (July-September) physical habitat time series for each life stage were calculated by coupling WUA-discharge curves with discharge time series. Mean summer WUA was calculated as the daily average for each life stage and year. Finally, physical habitat time series were transformed to carrying capacity time series by means of an allometric territory size relationship specifically developed for brown trout [35]: Log_10_ *T* = (2.64 – 0.96·*age category*)·Log_10_ *L* - (2.72 – 0.90·*age category*), where *T* (m^2^ of WUA) is territory size, *L* (cm) is length and *age category* is 0 for YOY trout or 1 for juvenile and adult trout. Carrying capacity was estimated for every age class (0+, 1+ and >1+), year and site through the following ratio: *K_i_* = *WUA_i_*/*T_i_*, where *K_i_* is the carrying capacity of age-class *i* (trout ha^−1^), *WUA_i_* is the mean summer WUA of age class *i* (m^2^ ha^−1^) and *T_i_* is the area of the territory used by an individual of average body size of age-class *i* (m^2^ trout^−1^).

### Water temperature modelling

We used the maximum mean water temperature during 7 consecutive days from July to September (*T_max7d-water_*, °C) to study potential limiting effects of physiological stress on brown trout performance. Seven days is the usual standard to estimate thermal tolerance of fish to short-term exposure (e.g., [19]). We developed a regional spatial model to predict extreme water temperatures in the study area during the study period (1993–2004). Since water temperature data were not available, they were estimated from air temperature data. At a first stage, we built a regional air temperature model by regressing annual maximum mean air temperature during seven consecutive days (*T_max7d-air_*) to latitude and altitude for 48 meteorological stations located at altitudes ranging from 38 to 1344 m a.s.l. Year was included as a random factor to induce an autocorrelation structure among data within the same year and account for yearly differences in the relationship among variables. *T_max7d-air_* was significantly related to latitude and altitude following the model: *T_max7d-air_* (°C) = 323.25 – 6.914·*Latitude* (decimal degree) - 0.0044·*Altitude* (m) (*R* ^2^ = 0.85, *P*<0.0001). At a second stage, we fitted a linear mixed-effects regression model relating *T_max7d-water_* to *T_max7d-air_* with river basin as a random factor. To do this, water temperature was recorded daily at study sites by means of data-loggers installed from June of 2004 to November of 2005. We employed *T_max7d-air_* as the independent variable since weekly averages of stream temperature and air temperatures are typically better correlated with each other than are daily values (e.g., [36]). The resulting model was highly significant (*R* ^2^ = 0.85, *P*<0.0001) and the within-basin fitted lines were specified by *T_max7d-water_*  =  3.372+0.656·*T_max7d-air_*, 4.688+0.589·*T_max7d-air_*, 4.171+0.626·*T_max7d-air_* for Aragón, Arga-Ega and Bay of Biscay basins, respectively.

### Data analyses

#### Effects of carrying capacity dynamics and competition on population size

We tested whether spatio-temporal variations in the number of individuals of a life stage (YOY, juvenile and adult) were driven by carrying capacity dynamics, levels of crowdedness (i.e., carrying capacity saturation) experienced by these individuals the previous year, and levels of crowdedness experienced by individuals of accompanying life stages. The level of carrying capacity saturation was measured as the relationship between observed density and estimated carrying capacity (*D/K* ratio) and was used as a proxy for intensity of competition among individuals. We also examined the effects of previous inter-seasonal and inter-annual limiting physical habitat bottlenecks on the performance of a life stage: (1) we used the average discharge (*Q_em_*) and the maximum mean discharge during 7 consecutive days (*Q_max7d_*) during emergence time (March-April) as proxies of physical habitat availability during this critical period. Both metrics were standardized by dividing by the historical median daily discharge to make them comparable among rivers significantly differing in discharge magnitude; (2) we used the relative carrying capacity ratio between two consecutive life stages to test whether the relative proportion of habitats available for a cohort along its ontogeny limits its performance. Density of life stage *x* at year *i* (*D_x,i_*), as response variable, was therefore regressed against predictors listed on Table 1. In the case of YOY trout, *D_0+,i_* was regressed against the level of carrying capacity saturation experienced by adult trout the previous year and the relative ratio between recruitment and adult stock carrying capacity. We fitted our regression models through the Random Forest algorithm (RF, [37]) implemented in the “randomForest” package [38] within the R environment [39].

**Table 1:** Predictors of YOY, juvenile and adult brown trout density used in Random Forest (RF) regression models.

Generic predictor	Predictors	Description
*K_x,i_*	*K_yoy_*, *K_juv_*, *K_adu_*	Carrying capacity of life stage *x* at year *i*
*D_x-1,i-1_/K_x-1,i-1_*	Past *D/K_yoy_*, *D/K_juv_*, *D/K_adu_*	Level of carrying capacity saturation experienced by individuals of age *x* on year *i*-1 when they were age *x*-1
*D_y,i_/K_y,i_*	*D/K_yoy_*, *D/K_juv_*, *D/K_adu_*	Level of carrying capacity saturation experienced by accompanying life stage *y* at year *i*
*K_x,i_/K_x-1,i-1_*	*K_yoy_/K_adu_*, *K_juv_/K_yoy_*, *K_adu_/K_juv_*	Relative carrying capacity ratio experienced by individuals of age *x* across years *i*-1 and *i*
*Q_em,i_*	*Q_em_*	Average discharge during emergence at year *i*
*Q_max7d,i_*	*Q_max7d_*	Maximum mean discharge during 7 consecutive days during emergence at year *i*

RF is a member of Regression Tree Analyses (RTA; [40]). RTA recursively partitions observations of the response variable into successive binary splits, each split being based on the value of a single predictor chosen through an exhaustive search procedure across all available predictors to minimize the unexplained variance of the response while maximizing the differences between the offspring branches. RF models increase prediction accuracy compared to traditional RTA by introducing random variation by growing each tree with a bootstrap sample of the training data and only using a small random sample of the predictors to define the split at each node. In outline, *ntree* bootstrap samples are randomly drawn with replacement from the training data, each containing 2/3 of the data (in-bag). Then, the RF algorithm searches the best split from a random subset of predictors (*mtry* variables from the whole set of variables) to construct the decision tree. Independent predictions (i.e., independent of the model-fitting procedure) for each tree are then made for the other 1/3 of the data that were excluded from the bootstrap sample (out-of-bag, or OOB). These predictions are averaged over all trees and the prediction error (OOB error) provides an estimate of the generalization error. Here, we first chose the optimal values of *ntree* and *mtry* that minimize the OOB error and then we proceeded to develop the RF model.

We employed RF models because they are free from distributional assumptions and automatically fit non-linear relationships and high-order interactions between predictors. Furthermore, as the number of trees increases, the generalization error always converges, so RF models cannot be over-fitted. Finally, as the OOB error is an unbiased estimate of the generalization error, it is not necessary to test the predictive ability of the model on an external data set [37]. The structure of the RF models can be examined using importance measures and partial dependence plots. Predictor Importance was assessed based on how much worse the OOB predictions can be if the values for that predictor are permuted randomly. The increase in mean of the error of a tree (mean square error, MSE) was used to measure the resulting deterioration of the predictive ability of the model after data permutation. Increase of MSE was computed for each tree and averaged over the forest (*ntree* trees). In addition, partial plots show the marginal effect of analyzed environmental variables in RF estimates of population size.

#### Effects of water temperature on population size

We deployed quantile regression (QR, [41]) to describe the limiting effect of water temperature on population size. We used this method because, contrarily to most regression analyses which focus exclusively on changes in the mean response, QR estimates multiple rates of change (slopes) in responses with unequal variation, so that it is especially suited to detect changes in heterogeneous distributions where other influencing factors are unmeasured and unaccounted for [42]. Importantly, QR allows the estimation of the rates of change near the upper and lower edges of responses, the parts of the distribution where limiting effects are typically detected. Therefore, we performed bootstrapped (1000 repetitions) QR estimates of quantiles using the “quantreg” package [43] within the R environment. We used the log-transformation of maximum mean water temperature during 7 consecutive days (*T_max7d-water_*) as the independent predictor of the residuals from the previously obtained random forest models [expressed as the log(*x*+1)-transformation of (observed density-predicted density)/predicted density], the response variable.

We additionally assessed the effects of water temperature on the temporal fluctuations of density within each sampling site. To do this, we fitted linear mixed effects models with the “lme4” package in R [44], using the same predictor and response variable and including site as a random factor (random intercept and slope) to induce a correlation structure between observations within the same site.

Finally, based on the calculated predictive water temperature regional model, we mapped *T_max7d-water_* for the average climate conditions during the study period using ArcGis 9.2 software (ESRI Inc., Redlands, CA, USA). We implemented subsequently the linear mixed effects models previously obtained to map the spatially-explicit distribution of average population thermal carrying capacity across the region during the study period. We eventually projected the amount of thermal suitable habitat (*T_max7d-water_* equal or below 19.4°C; see [24]) and the thermal carrying capacity under warming scenarios based on the air temperature regional projections for the B2 SRES emission scenario presented by Brunet et al. [45].

## Results

### Effects of carrying capacity dynamics and competition on population size

**Figure 1:**
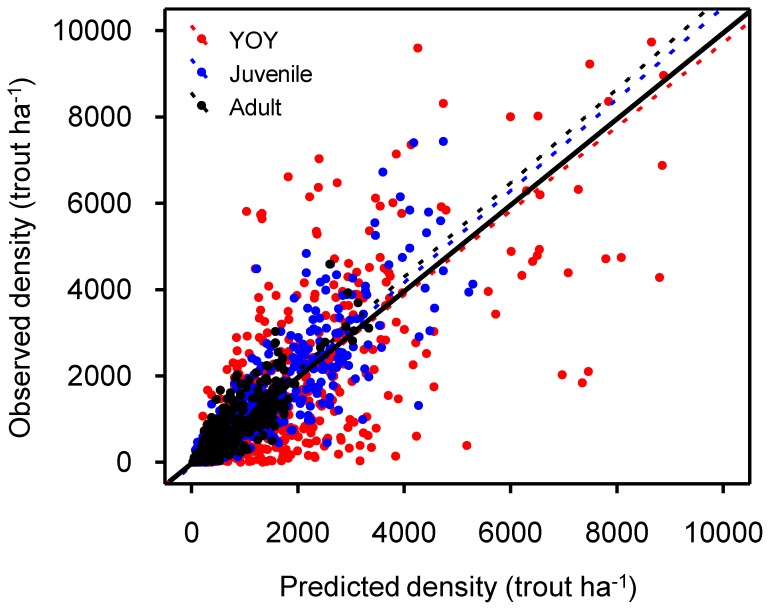
Random Forest performance on density of each life stage. Observed vs. predicted density of YOY, juvenile and adult brown trout. Dotted lines represent fitted linear models (YOY: *y* = 13.15+0.96·*x*, *R* ^2^ = 0.50, *P*<0.001; Juvenile: *y* = −83.37+1.04·*x*, *R* ^2^ = 0.75, *P*<0.001; Adult: *y* = −56.56+1.06·*x*, *R* ^2^ = 0.76, *P*<0.001) while solid line shows perfect match between observed and predicted.

The out-of-bag estimates of the error rate (OOB error) were used to select the optimum Random Forest parameters (*mtry* = 3, *ntree* = 600 for all models). Compared to older life stages, RF performance was subordinate for YOY trout, in which the model only explained 50% (*P*<0.001) of the observed density variance (Fig. 1). The RF algorithm performed better for juvenile and adult life stages with the models explaining 75% and 76% (*P*<0.001) of total density variance. OOB predictions seemed to be in proper scale (regression slopes ranging from 0.96 to 1.05∼1) with slight deviations from observed data (Fig. 1).

**Figure 2:**
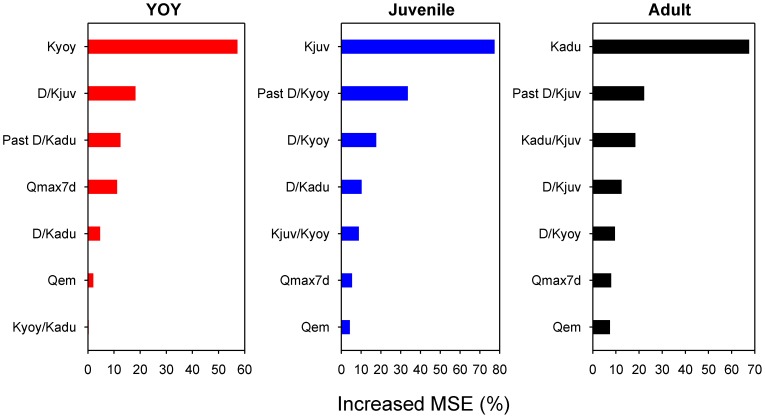
Predictor importance on density of each life stage. The plots show predictor importance measured as the increased mean square error (%), which represents the deterioration of the predictive ability of the model when the data of a predictor are randomly permuted. Higher Increased MSE indicates greater predictor importance. Note that axes are not constant across plots.

**Figure 3:**
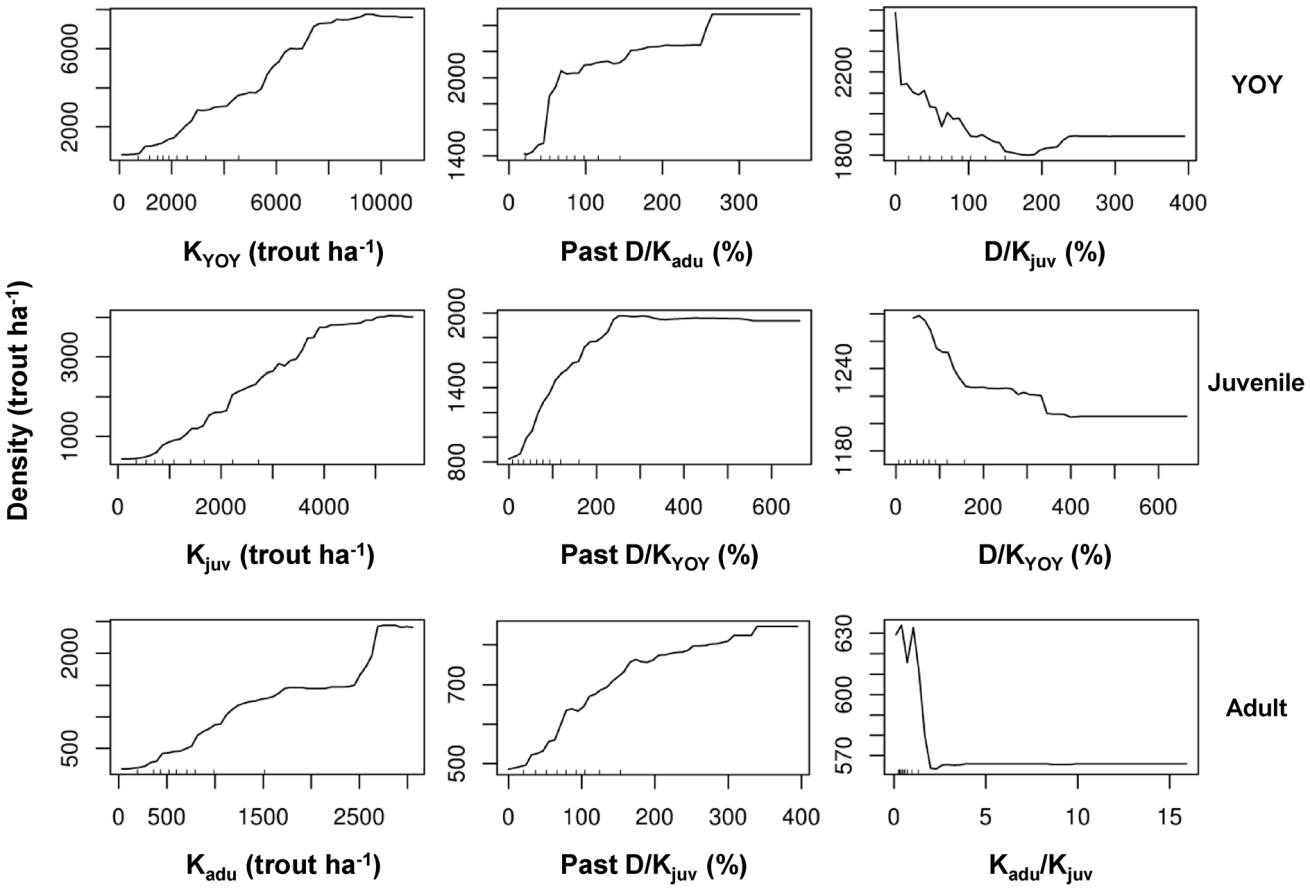
Marginal contribution of most important predictors on density of each life stage. Partial plots representing the marginal contribution of the three most important predictors in the RF models to density of each life stage while averaging out the effect of all the other predictors. Note that in a partial plot of marginal effects, only the range of values (and not the absolute values) can be compared between plots of different predictors.

Carrying capacity (*K*) ranked first in importance for all life stages, its contribution to the prediction accuracy of the models being disproportionately higher than the rest of predictors (Fig. 2). Carrying capacity saturation experienced the previous year (past *D/K* ratio) was an important predictor of density for all life stages. It had considerable importance for juveniles, but appeared less important for YOY trout, whose density variations were strongly driven by carrying capacity (Fig. 2; note also the marked differences in the range of predicted density values across predictors shown in Figure 3). Density of a life stage increased with increasing past *D/K* ratio up to a threshold where further increases in *D/K* ratio have either deleterious or no effects on density (Fig. 3). YOY and juvenile trout interacted in an antagonistic manner as density of either life stage decreased with increasing *D/K* ratio of the other one (Fig. 3). Intercohort interactions did not contribute noticeably to adult trout density though, since neither *D/K_yoy_* nor *D/K_juv_* ranked among its three most important predictors (Fig. 2). By contrast, the relative ratio between adult and juvenile *K* was a top-three determinant of adult density (Fig. 2), with maximum performance at a relative ratio close to one and a sharp drop at values greater than two (Fig. 3). Interestingly, *Q_max7d_* was only important for YOY trout (Fig. 2), density falling sharply when *Q_max7d_* exceeded the historical median daily discharge and the highest negative effects being observed during strong flow events when *Q_max7d_* values were over ten times this historical median daily discharge (plot not shown).

### Effects of water temperature on population size

**Figure 4:**
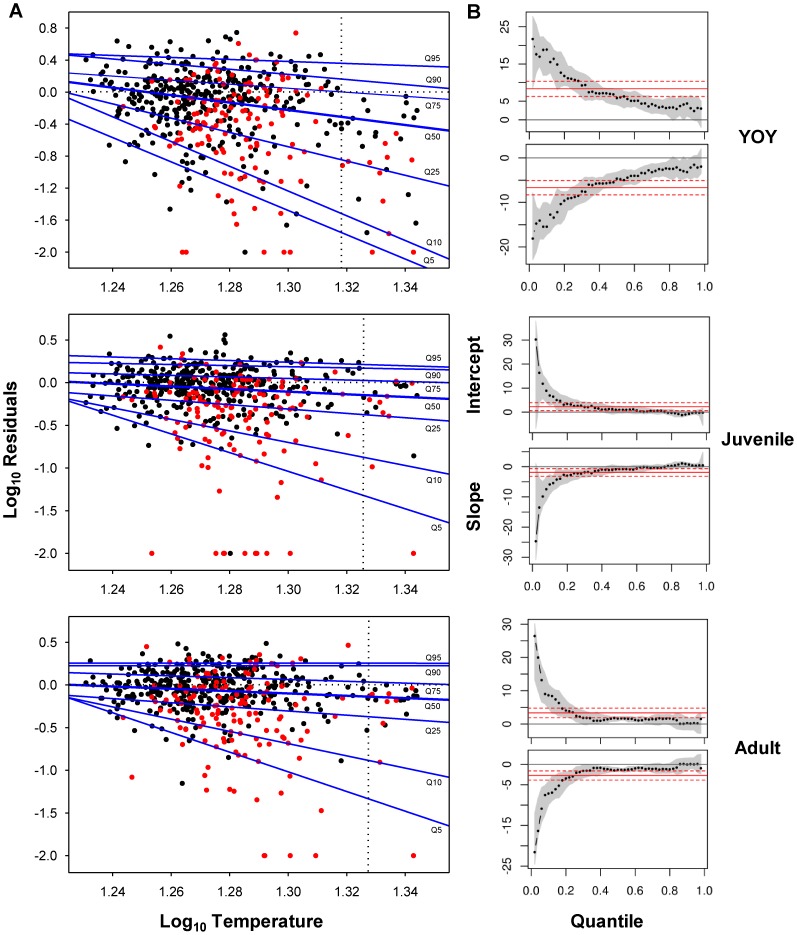
Limiting effects of water temperature on density of each life stage. (A) Quantile Regression (QR) estimates of the 5^th^, 10^th^, 25^th^, 50^th^, 75^th^, 90^th^ and 95^th^ quantiles (*Q5*, *Q10*, *Q25*, *Q50*, *Q75*, *Q90* and *Q95*) of log(*x*+1)-transformed residuals from RF models vs. log-transformed maximum mean water temperature during 7 consecutive days. Horizontal dotted lines show perfect match between observed and predicted density from RF models. Vertical dotted lines indicate the critical temperature threshold (CTT) beyond which no positive residuals exist. Red data belong to sampling sites having the lowest mean carrying capacity (below the 25^th^ percentile of the whole distribution); (B) Intercept and slope coefficient estimates with associated confidence intervals for QR across varying quantiles. Mean and confidence interval of the mean are represented in red.

Regression quantiles for YOY trout were significant up to the *Q95*, negative deviation from RF model's predicted values increasing with increasing *T_max7d-water_* throughout the whole range of quantiles (Fig. 4A). Slope of the regression quantiles significantly differed across quantiles, lower quantiles having increasingly greater negative slopes (Fig. 4B). By contrast, regression quantiles for juveniles and adults were only significant up to the *Q75* and regression slopes were not significantly different down to the *Q25* where negative steepness of slopes significantly increased with lower quantiles (Fig. 4B). Regression slopes for YOY were significantly steeper for any quantile compared to juvenile and adult models (Fig. 4B). All statistical outputs from quantile regressions can be checked in Appendix S1.We observed the existence of a critical temperature threshold (CTT) beyond which no positive residuals existed, and this threshold increased with age, from 1.318 (20.8°C) for YOY to 1.330 (21.4°C) for adult trout (Fig. 4A). Further analyses of the residuals' distribution revealed that most of the data linked to the lowest quantiles (*Q5*–*Q25*) belonged to the sampling sites having also the lowest mean *K* (lower than the 25^th^ percentile of the *K* distribution across the whole population of sites) (Fig. 4A). Finally, residuals from the most limiting quantile (*Q5*) were significantly related to anthropogenic disturbance metrics (Appendix S2).

**Figure 5:**
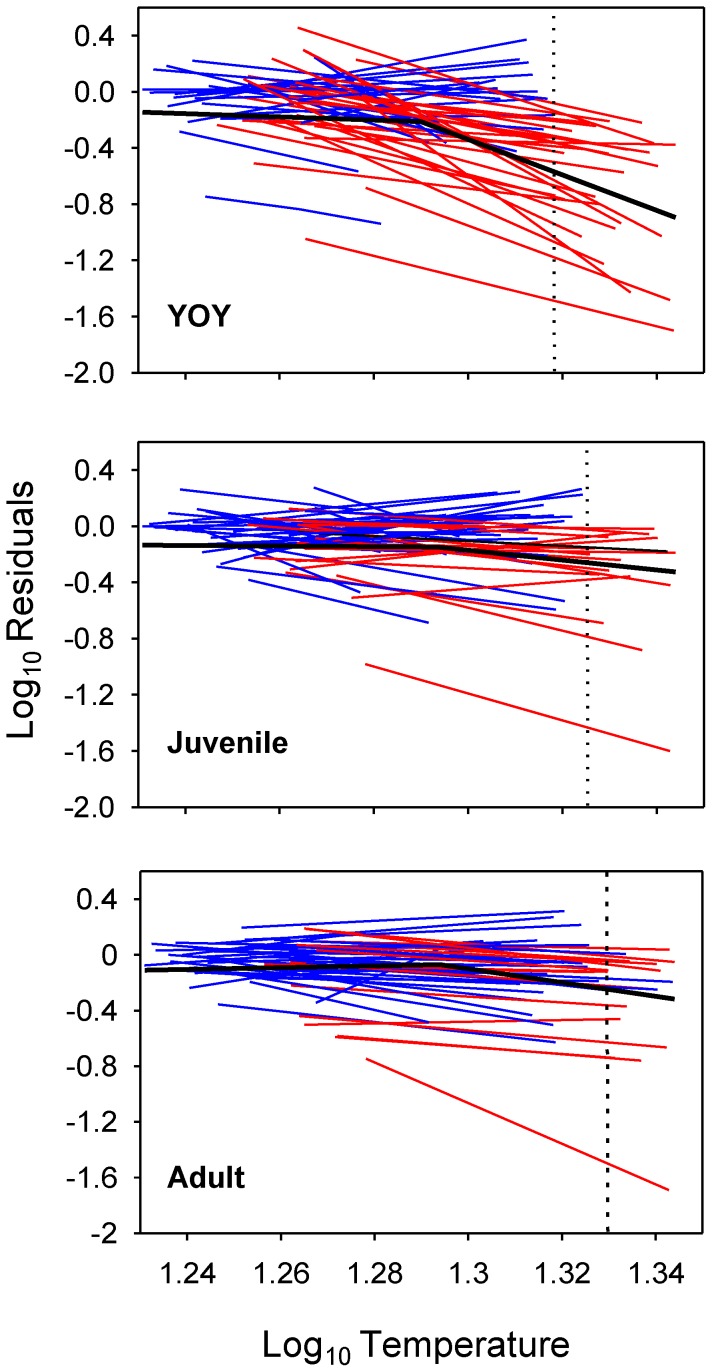
Regression lines for site-specific water temperature vs. density temporal relationships. Red/blue lines are fitted models for sites whose temperature range did include/not include values over the critical temperature threshold (CTT; vertical dotted lines). Piecewise linear mixed-effects regression models for the whole population of sampling sites are shown in black.

Focusing on temporal trends, we observed that regression lines between temperature and YOY density in sites whose temperature range did not include values beyond the CTT could adopt different patterns, having either positive, negative or no slope (Fig. 5). Nevertheless, there was always a negative relationship between temperature and density in sites where temperature was over the CTT at least at one year during the study period. This pattern was accurately described through a piecewise linear mixed-effects regression model, comprising a non-significant line with a population slope of −1.12 (*N* = 509, *t* = −0.68, *P* = 0.50) and significant random variation across sites (*SD* = 3.89), plus a highly significant negative line with a population slope of −12.80 (*N* = 509, *t* = −5.22, *P*<0.001) allowing for high random variation around it (*SD* = 9.21) (Fig. 5). This pattern was less marked for juveniles and adult trout. The slope of the juvenile's regression model after the breakpoint was not significant (slope = −1.28, *SD* = 9.17; *N* = 509, *t* = −1.28, *P* = 0.20), while adult's one was in the boundary of significance (slope = −2.94, *SD* = 2.39; *N* = 509, *t* = −2.11, *P* = 0.035) (Fig. 5). Interestingly, the breakpoint was almost the same for the three models (around 19.4°C). When aggregating all life stages together (population model), total population density was not significantly related to temperature (slope = 1.27, *SD* = 2.87; *N* = 509, *t* = 1.39, *P* = 0.16) up to 19.42°C when it significantly declined at a rate of −4.11 (*N* = 509, *t* = −2.51, *P* = 0.016) with high random variation in regression slopes across sites (*SD* = 6.86).

**Figure 6:**
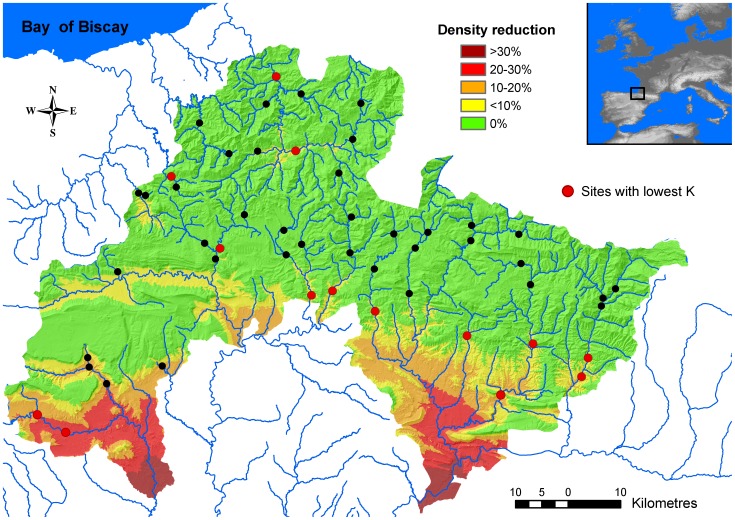
Spatial patterns of density reduction set by the thermal capacity of the environment. Spatially-explicit temperature-driven decrease in potential population numbers predicted by RF model. Effects of temperature on density are estimated from the piecewise linear mixed-effects regression models for the whole population of sampling sites. Dots represent sampling sites, while red dots represent those sites having the lowest mean carrying capacity (below the 25^th^ percentile of the whole distribution: K<2700 trout ha^−1^).

Spatial simulations based on the piecewise regression population model and temperatures averaged for the study period showed that the thermal capacity of the environment was permanently lower than its habitat capacity (up to a 36%) in a significant area of the study region (Fig. 6). Projected suitable thermal habitat will decrease down to 7% of total study area by the year 2100 under the B2 SRES emission scenario. By that time and emission scenario, population thermal capacity will be on average 39.9±10.0% (range 1.5–68.1) lower than its habitat capacity, but YOY maximum potential density will be reduced on average a 61.3±19.1% (range 2.4–93.1).

## Discussion

Recent human-induced species' extinction rates are overwhelmingly greater than at any other time in human history [46] and number of species at the verge of imminent extinction is also increasing at an unparalleled speed [47]; meanwhile, current rates of population extirpation are at least three orders of magnitude higher than species extinction rates [48]. This latter is made evident by the fact that species are shifting their ranges two or three times faster than previously reported [20], especially freshwater fish, which may be responding to global warming at higher rates than terrestrial organism [49]. However, significant population declines of species of high conservation concern may occur before any reduction in range is observed, so that determining and modelling the factors driving population size and trends is crucial to predict their future extinction risk [50]. In our study, distribution and dynamics of carrying capacity along with emergent density-dependent responses explained up to 76% of spatio-temporal density variability of juvenile and adult brown trout, but only 50% of YOY's. By contrast, YOY trout were highly sensitive to thermal conditions, their performance declining with increasing temperature at a higher rate than older life stages, and disruptions being triggered at lower temperature thresholds.

Carrying capacity (*K*), primarily based on quantity and quality of available physical habitat, was the strongest and most consistent contributor to density of any life stage; by contrast, most of analyzed habitat and competition predictors just qualified final numbers within narrow ranges around set carrying capacity. This provides empirical support to the theoretical prediction that density-independent factors should predominate over density-dependent ones in setting population numbers when environmental conditions are harsh - such as the ones experienced in distributional margins - [51]. Not only present physical habitat conditions but also previous habitat bottlenecks limited density though. Strong flow events during emergence depressed summer recruitment. Such disturbance events drastically reduce the quantity and quality of suitable physical habitat, which results in high YOY mortality through both direct downstream displacement of subordinate individuals without shelter (e.g., [52]) and delayed carry-over effects on individuals occupying low-quality habitats that affect their performance in the following season (see [53]). We also observed that juvenile physical habitat can limit subsequent adult abundance. Halpern et al. [54] showed that, in stage-structured species, juvenile habitat availability limits adult abundance in a relatively small region of parameter space compared with the regions where recruitment and adult *K* are limiting. This notion appears to apply for our populations since limitations in adult abundance by juvenile physical habitat seemed to be critical only in populations with low adult *K*.

Physical habitat quality and quantity is also a resource that, by limiting *K*, clearly stimulates the operation of density dependence. Intracohort density dependence was the second most important and consistent density predictor, having a large effect on final numbers. The annual realized density of a cohort relative to its *K* increased with increasing level of *K* saturation experienced by the cohort the previous year (or by adult stock in the case of YOY). This is in accordance with many model systems which suggest that individuals are strongly affected by both current and past environments, even when the past environments may be in previous generations (reviewed by Benton et al. [8]). This intracohort response is non-linear so that beyond a saturation level further increases in cohort crowdedness have deleterious or no effects on cohort numbers next year. The saturation threshold is well over 100%, indicating that a large proportion of individuals may remain in the stream as non-territorial or floaters. This is consistent with the idea that most animal populations spend more time above than below carrying capacity since population regulation is generally the result of a concave relationship between a population's growth rate and its size [10] (but see [55]). The intracohort density dependence also implies that density disruptions can be transmitted through generations so that constant pressures (either natural or anthropogenic) over time on a population may substantially depress its growth rate, and thus its density at equilibrium, turning the population more prone to become extinct through stochastic events [3]. Furthermore, we found that YOY and juvenile densities were mutually affected by the level of crowdedness experienced by the competing cohort, suggesting a negative density-dependent regulation of each life stage over the other. In stage-structured populations, density-dependent interactions between life stages can affect population trajectories and lead to natural selection operating within populations and across life stages (see [56] and references therein). Density-dependent processes may interact in fact with density-independent factors (for e.g., *K* dynamics) in shaping adaptive landscapes, potentially leading to strong non-additivity in the development of vital rates driving population dynamics [57].

Distribution and abundance of species reflect their specific traits that allow them to pass through multiple environmental filters at hierarchical spatial scales, so that species lacking traits suitable for passing through a large scale filter are limited in abundance at all lower scales [58]. In our study, high summer temperatures restricted or reduced brown trout habitat use from certain areas of study basins where the physical microhabitat was otherwise suitable (see Fig. 6). Our quantile regression models showed that water temperature had a limiting effect on density, this limitation being significantly stronger for YOY trout. This was expected as small fish are more sensitive to temperature fluctuations than larger fish (see [19] for details of underlying mechanisms). Importantly, regression slopes significantly changed across quantiles, the steepest slopes being associated to the lowest quantiles. This means that rising temperatures had an increasingly higher negative effect on density performance as density departs from the maximum potential numbers set by *K* and density-dependent dynamics. There is a gradual shift from physical habitat to temperature being the active environmental limiting factor.

The reasons of the shift could be two-fold. First, such changes in regression slopes indicate strong interactions of temperature with unmeasured factors while results reported in Appendix S2 reveal complex synergies among temperature and multiple anthropogenic drivers and stressors. The tight significant relationship between the density-carrying capacity ratio and levels of anthropogenic disturbance previously observed in most of the study populations [28] suggests that the degree of mismatch between densities observed and predicted from random forest models (RF) would be driven by disturbance intensity. In such a case, increasing disturbance would result in physical habitat conditions being no longer an active limiting factor so that density dynamics may get decoupled from *K* dynamics. On the contrary, the negative effects of increasing temperatures are stronger in populations already disrupted by anthropogenic stressors. Further, temperature impacts on density would be synergistically amplified by disturbance intensity since the predominant anthropogenic drivers in the study area (agricultural land uses and damming) typically imply both a local increase in water temperature and a decrease in energy inputs [59]–[61], which would affect accordingly fish energy budgets. Second, our data indicate that there is a critical temperature threshold (CTT) beyond which observed density is always lower than predicted by RFs irrespective of disturbance intensity. This CTT, beyond which the thermal capacity of the environment is always lower than its habitat capacity, decreases with age and roughly matches the incipient thermal limit for survival estimated for the different brown trout life stages (see [19]). This CTT is likely to diminish with increasing levels of anthropogenic disturbance, a clear example of how synergies among stressors form self-reinforcing mechanisms that hasten the dynamics of population extinction [2].

The analysis of temporal trends within sampling sites was consistent with such a picture. There is a thermal range within which there are strong spatial variations across populations in the functional relationship between temperature and density fluctuations, the sign of the relationship being dependent on anthropogenic disturbance intensity. However, there is a point beyond which density performance always decline with increasing temperature and at a faster rate than before. This pattern is especially patent in YOY trout, but less marked in older life stages. The breakpoint is however fairly constant across life stages (around 19.4°C), matching the upper thermal limit for feeding, where the starvation zone begins for brown trout (see [19]). This differentiation is important since it entails that population decline may start well below the CTT. In general, individuals without territories may survive either adopting a high-return/high-cost strategy, attempting to maximize energy intake at a cost of increasing interactions with territorial individuals, or a low-return/low-cost strategy, occupying poor feeding positions but minimizing energy costs by avoiding competition [62]. Within the starvation zone, the high-return/high-cost strategy rapidly fails and with increasing temperatures the low-return/low-cost strategy is no longer energetically feasible either. Over the CTT, mortality of individuals holding a territory in high-quality habitat patches could not be buffered by the floater population anymore, so that the population may turn unstable over time.

Two natural compensatory responses are possible against anthropogenic global warming. Given enough time and dispersal capabilities, species may shift to more favourable thermal environments, or they may track climate change through adaptation to avoid demographic collapse and extinction [21]. However, the probability of evolutionary rescue seems to be contingent on low rates of environmental deterioration [63] and there is no empirical evidence of thermal adaptation at the upper temperature limits for either survival or feeding in salmonids [19], [64]. We have also provided evidence that anthropogenic disturbances may fasten the rate of population decline under warming, while the vast network of dams in our study basins (see [28]) would additionally prevent upward dispersal to find suitable thermal conditions. Based on our temperature models, we predict that the 93% of our study area would be thermally unsuitable for brown trout and the thermal capacity of the environment for recruitment could be on average 61% lower than its carrying capacity by 2100 under the ecologically friendly B2 SRES emission scenario. In that case, recruitment disruptions would have long-term amplifying downstream effects through density-dependent processes. This is dramatic as populations with the lowest *K* are located in areas where thermal constraints are predicted to be highest (Fig. 6), so that they are likely to become extinct well before 2100. It is worth noticing that this modelling exercise is somehow burdened by uncertainties inherent to both habitat suitability and climate envelope models (for e.g., see [65]–[66] for further discussion). Notwithstanding possible uncertainties, for marginal salmonid populations constrained to linear networks, temperature is destiny in a warming world [67]. Drastic reductions of distributional ranges are projected even in core areas [68]. By contrast, Piou and Prévost [69] model predicts that rising river temperatures alone should not lead open anadromous populations to extinction and that such river warming may even bumper the synergistic negative effects of flow regime alteration and ocean conditions deterioration on population persistence.

We acknowledge that this is an oversimplified picture as population trajectories of individual species cannot be scrutinized in isolation considering that climate change can alter multitrophic level interactions so strongly that entire food webs can undergo radical restructuring [70]–[71]. Warming should lead to a decrease in *K* and/or a decrease in the mean body mass (to buffer the potential decrease in *K*) if altered conditions cannot concurrently increase the supply rate of a species' feeding resources [72]. Nevertheless, notwithstanding that our predicted numbers are certainly uncertain, they roughly portray the gloomy fate of thermally-sensitive species occurring at contracting range margins under limited potential for adaptation and/or dispersal.

## Supporting Information

Appendix S1 **Quantile regressions statistical output.**
(DOC)

Appendix S2 **Effects of anthropogenic stressors on population size.**
(DOC)
