# Supplementary material for: Thermal Carrying Capacity for a Thermally-Sensitive Species at the Warmest Edge of Its Range
Source: PLoS One. 2013 Nov 25;8(11):e81354. doi: 10.1371/journal.pone.0081354 (PMC3840006; doi:10.1371/journal.pone.0081354)
Supplement: Appendix S1 — Quantile regressions statistical output. (DOC) [file pone.0081354.s001.doc]

# Thermal carrying capacity for a thermally-sensitive species at the warmest edge of its range

# ONLINE SUPPLEMENTARY MATERIAL

# Appendix S1: Quantile regressions statistical output

**Table S1.** Quantile Regression estimates of log(*x*+1)-transformed residuals from RF models vs. log-transformed maximum mean water temperature during 7 consecutive days. Estimates of regression slopes (standard error) and their probabilities (ns = non-significant, **P*<0.05, ***P* <0.01, ****P* <0.001) are shown.

| Quantile | YOY | Juvenile | Adult |
| --- | --- | --- | --- |
| *Q5* | Slope=-15.18 (2.85), *t*=-5.31 *** | Slope=-10.97 (4.66), *t*=-2.35 * | Slope=-11.52 (3.34), *t*=-3.45 *** |
| *Q15* | Slope=-13.01 (2.01), *t*=-5.37 *** | Slope=-4.72 (1.78), *t*=-2.46 ** | Slope=-5.52 (1.67), *t*=-3.17 ** |
| *Q25* | Slope=-8.92 (1.86), *t*=-4.79 *** | Slope=-2.54 (1.11), *t*=-2.64 ** | Slope=-2.42 (0.94), *t*=-2.77 ** |
| *Q35* | Slope=-6.27 (1.51), *t*=-4.14 *** | Slope=-2.42 (0.81), *t*=-3.12 ** | Slope=-1.47 (0.69), *t*=-2.39 * |
| *Q50* | Slope=-4.66 (0.98), *t*=-5.31 *** | Slope=-1.54 (0.61), *t*=-2.49 * | Slope=-1.36 (0.55), *t*=-2.48 * |
| *Q65* | Slope=-3.43 (0.92), *t*=-3.69 *** | Slope=-1.20 (0.54), *t*=-2.23 * | Slope=-1.27 (0.44), *t*=-2.28 * |
| *Q75* | Slope=-2.97 (0.85), *t*=-2.50 * | Slope=-0.99 (0.41), *t*=-2.10 * | Slope=-1.06 (0.60), *t*=-1.60 ns |
| *Q85* | Slope=-2.52 (1.15), *t*=-2.9 * | Slope=-0.64 (0.77), *t*=-0.57 ns | Slope=-0.81 (0.78), *t*=-1.03 ns |
| *Q95* | Slope=-1.25 (1.44), *t*=-0.86 ns | Slope=-0.79 (0.49), *t*=-1.25 ns | Slope=-0.01 (0.43), *t*=-0.03 ns |

**Table S2.** Test for equality of slopes for fitted regression quantiles (ANOVA: ns = non-significant, **P*<0.05, ***P* <0.01, ****P* <0.001).

| **Quantile range** | ***F* value** | ***P*** |
| --- | --- | --- |
| YOY |  |  |
| *Q5*-*Q95* | *F*_8,4510_=3.47 | <0.001 *** |
|  |  |  |
| Juvenile |  |  |
| *Q25*-*Q95* | *F*_6,3487_=0.83 | 0.55 ns |
| *Q5*-*Q25* | *F*_2,1495_=3.20 | 0.041 * |
|  |  |  |
| Adult |  |  |
| *Q35*-*Q95* | *F*_5,3049_=1.23 | 0.29 ns |
| *Q5*-*Q35* | *F*_3,2033_=5.17 | 0.001 ** |
|  |  |  |

**Table S3.** Test for equality of slopes for fitted regression quantiles across life stages (two-tailed t-test: ns = non-significant, **P*<0.05, ***P* <0.01, ****P* <0.001).

| Quantile | YOY vs. Juvenile | YOY vs. Adult | Juvenile vs. Adult |
| --- | --- | --- | --- |
| *Q5* | *t*=0.78, *P*= 0.44 ns | *t*=0.84, *P*= 0.40 ns | *t*=-0.10, *P*= 0.92 ns |
| *Q15* | *t*=3.08, *P*= 0.002 ** | *t*=2.87, *P*= 0.004 ** | *t*=-0.33, *P*= 0.74 ns |
| *Q25* | *t*=2.97, *P*= 0.003 ** | *t*=3.11, *P*= 0.002 ** | *t*=0.08, *P*= 0.94 ns |
| *Q35* | *t*=2.24, *P*= 0.025 * | *t*=2.89, *P*= 0.004 ** | *t*=0.90, *P*= 0.37 ns |
| *Q50* | *t*=2.65, *P*= 0.008 ** | *t*=2.88, *P*= 0.004 ** | *t*=0.21, *P*= 0.83 ns |
| *Q65* | *t*=2.08, *P*= 0.038 * | *t*=2.12, *P*= 0.034 * | *t*=-0.10, *P*= 0.92 ns |
| *Q75* | *t*=2.31, *P*= 0.021 * | *t*=1.99, *P*= 0.047 * | *t*=-0.10, *P*= 0.92 ns |
| *Q85* | *t*=1.37, *P*= 0.17 ns | *t*=1.23, *P*= 0.22 ns | *t*=-0.15, *P*= 0.88 ns |
| *Q95* | *t*=0.31, *P*= 0.76 ns | *t*=0.82, *P*= 0.41 ns | *t*=0.71, *P*= 0.48 ns |
