# Supplementary material for: Thermal Carrying Capacity for a Thermally-Sensitive Species at the Warmest Edge of Its Range
Source: PLoS One. 2013 Nov 25;8(11):e81354. doi: 10.1371/journal.pone.0081354 (PMC3840006; doi:10.1371/journal.pone.0081354)
Supplement: Appendix S2 — Effects of anthropogenic stressors on population size. (DOC) [file pone.0081354.s002.doc]

# Thermal carrying capacity for a thermally-sensitive species at the warmest edge of its range

# ONLINE SUPPLEMENTARY MATERIAL

# Appendix S2: Effects of anthropogenic stressors on population size

**Objectives in Appendix S2**

# A major result from quantile regression analyses was that regression slopes significantly changed across quantiles, the steepest slopes being associated to the lowest quantiles. This means that increasing temperatures have a stronger negative effect on density performance as density deviates from the maximum potential numbers predicted by random forest (RF) models. Such changes in regression slopes indicate strong interactions of temperature with factors which were not taken into account in the analyses. We hypothesize that temperature effects on population performance would be stronger in populations already weakened by anthropogenic stressors. If so, residuals from regression quantiles must be significantly correlated to disturbance metrics.

# The “greater temperature effects on populations already disrupted” pattern must be also reflected in the temporal dimension, that is, when analysing temporal trends in population size within sites. If the pattern is true, the negative slope of the relationship between temperature and residuals from RF models must be steeper in populations showing lower performance (higher deviation from densities predicted by RF models).

**Methods in Appendix S2**

*Anthropogenic stressors*

We measured a total of 24 metrics to quantify the degradation levels derived from different human activities operating at diverse spatial scales (Table S4 in Appendix S2). Two approaches were used in this characterization: (1) *in situ* measures to describe local characteristics at each sampling site; and (2) remotely collected data, using geographic information systems (GIS) to describe attributes at larger spatial scales. Hence, degradation metrics measured at the watershed and segment spatial scales were calculated by means of ArcGis 9.2 software (ESRI Inc., Redlands, CA) from digital data provided by regional agencies. Land use metrics were measured at three spatial scales: local restricted to the riparian zone (defined as a 100 m buffer at each river side), complete river network restricted to the riparian zone, and watershed (within the whole catchment area). Water quality data were obtained from regional datasets. Angling statistics were calculated from data derived from creel-surveys conducted by regional agencies during the fishing season (March-August) along the study period. The invasive fish species richness index, which combines abundance and number of invasive species, was reckoned from sampling data collected by regional agencies.

**Table S4.** Anthropogenic degradation metrics used to characterize the sampling sites.

| Spatial scale | Variable | Description (units) |
| --- | --- | --- |
| Watershed | Urban_N_ | % of urban land use within the riparian zone along the complete river network upstream the study site |
|  | Cultivated_N_ | % of cultivated land within the riparian zone along the complete river network upstream the study site |
|  | Pasture_N_ | % of pastures within the riparian zone along the complete river network upstream the study site |
|  | Urban_W_ | % of urban land use within the watershed upstream the study site |
|  | Cultivated_W_ | % of cultivated land within the watershed upstream the study site |
|  | Pasture_W_ | % of pastures within the watershed upstream the study site |
|  | Quarries | Number of quarries within the watershed upstream the study site (n 1000 ha^-1^) |
|  | Mines | Number of mines within the watershed upstream the study site (n 1000 ha^-1^) |
|  | Industrial premises | Number of industrial premises within the watershed upstream the study site (n 1000 ha^-1^) |
|  | Upstream dams | Number of dams upstream the study site |
|  | Impassable upstream dams | Number of impassable dams upstream the study site |
|  | Downstream dams | Number of dams downstream the study site |
| Segment | Urban_L_ | % of urban land use within the riparian zone along the study segment |
|  | Cultivated_L_ | % of cultivated land within the riparian zone along the study segment |
|  | Pasture_L_ | % of pastures within the riparian zone along the study segment |
|  | Altered flow | % of the study segment presenting an altered flow regime |
| Site | Dissolved oxygen | Concentration (O_2_, mg l^-1^) |
|  | pH | pH value |
|  | Nitrites | Concentration (NO_2_^-^, mg l^-1^) |
|  | Ammonia | Concentration (NH_4_^+^, mg l^-1^) |
|  | Phosphate | Concentration (PO_4_^3-^, mg l^-1^) |
|  | Annual harvest | Angling annual harvest (Trout ha^-1^ year^-1^) |
|  | Exploitation rate | Angling exploitation rate (%) |
|  | Invasive fish species richness | Index combining number and abundance of invasive fish species present at the study site along the study period |

*Data analyses*

We tested whether the spatial variance in population size unexplained by environmental factors was correlated to anthropogenic degradation metrics. To do this, we first averaged per sampling site the residuals from the most limiting regression quantile (*Q5*), which was used as the dependent variable. We then fitted linear mixed effects models with the “nlme” package in R (Pinheiro et al. 2011). Prior to regression analyses, we performed correlation analyses to eliminate highly correlated (*r*>|0.7|) degradation metrics in order to avoid multicollinearity. We included basin as a random factor to induce a correlation structure between observations within the same basin, since sources and levels of degradation varied among basins. We first tried to find the optimal random structure and then looked for the optimal fixed structure by performing sequential removal of non-significant fixed effects and subsequent model comparisons using log-likelihood ratios according to the procedure recommended by Zuur et al. (2009).

Secondly, we tested whether the slope of the regression line between temperature and residuals from RF models was significantly related to the position of the line within the cloud of points. To do this, we fitted a General Regression Model (GRM), for each life stage, with the slope of the regression line as the response variable and residuals from the RF model (averaged for the whole study period) and maximum *T_max7d-water_* experienced during the whole study period as predictors. Predictors were log-transformed. The significance level for all statistical tests was set at α = 0.05.

**Results in Appendix S2**

The best explaining model for YOY trout had a random structure comprising a random intercept and random slopes for nitrites and cultivated land, while fixed effects were nitrites, cultivated land, pastures and upstream dams (Table S5 in Appendix S2). Examination of parameter coefficients showed that negative effects of nitrites and cultivated land on density were only significant at specific basins. Best-supported models for juveniles and adults presented the same random structure, including a random intercept and random slopes for cultivated land and upstream dams, and fixed structure, consisting of cultivated land, upstream dams and exploitation rate (Table S5 in Appendix S2). As observed with YOY trout, the population slope for cultivated land was not significantly different from zero so that increasing proportion of land used for cultivation impacted density only at specific basins. The same pattern was observed for juvenile trout regarding number of upstream dams. Note that all stressors are negatively related to mean residuals because these are referred to the lowest (and most limiting) temperature quantile, so that increasing residuals indicate better density performance at a given temperature.

**Table S5.** Parameter estimates from the linear mixed effects models with basin as a random effect (intercept and slope) that best explain variation in mean residuals from the most limiting regression quantile (*Q5*) by life stages.

| **Response variable** | **Parameters** | **Coefficients (SE)** | **t** |
| --- | --- | --- | --- |
| Mean Residuals YOY | (Random intercept) | 0.028 (0.248)* |  |
|  | (Random slope Nitrites) | 0.066 (0.248)* |  |
|  | (Random slope Cultivated_N_) | 0.112 (0.248)* |  |
|  | Intercept | 0.868 (0.040) | 21.89 |
|  | Nitrites | -0.027 (0.025) | -1.05 |
|  | Cultivated_N_ | -0.088 (0.081) | -1.08 |
|  | Pasture_N_ | -0.097 (0.039) | -2.50 |
|  | Upstream dams | -0.179 (0.038) | -4.74 |
| Mean Residuals Juvenile | (Random intercept) | 0.036 (0.162)* |  |
|  | (Random slope Cultivated_N_) | 0.122 (0.162)* |  |
|  | (Random slope Upstream dams) | 0.107 (0.162)* |  |
|  | Intercept | 0.596 (0.034) | 17.35 |
|  | Cultivated_N_ | -0.094 (0.077) | -1.23 |
|  | Upstream dams | -0.089 (0.076) | -1.18 |
|  | Exploitation rate | -0.054 (0.026) | -1.92 |
| Mean Residuals Adult | (Random intercept) | 0.178 (0.214)* |  |
|  | (Random slope Cultivated_N_) | 0.010 (0.214)* |  |
|  | (Random slope Upstream dams) | 0.175 (0.214)* |  |
|  | Intercept | 0.593 (0.107) | 5.53 |
|  | Cultivated_N_ | -0.033 (0.036) | -1.37 |
|  | Upstream dams | -0.221 (0.107) | -1.98 |
|  | Exploitation rate | -0.075 (0.034) | -2.01 |

* Estimated coefficients for intercepts and slopes are SD of the random parameters, with SD of residual variation given in parentheses

Note: Only significant parameters are included in the final models

The slope of the regression line between maximum temperature and residuals from RF models for all life stages was significantly related to maximum *T_max7d-water_* experienced during the whole study period, residuals from the RF model (averaged for the whole study period) and their interaction (Table S6 in Appendix S2). According to the models, the negative effects of temperature on population performance increased with increasing population disruption. There is a strong interaction between both predictors. When the range of temperature fluctuations is within the tolerance window (low maximum temperatures), increasing temperatures would have a positive effect on population performance in healthy populations (low deviation from maximum potential numbers set by RF models). This is true up to a threshold. After such threshold, rising maximum temperatures will have increasingly negative effects even in healthy populations. Since the effect of temperature is qualified by population disruption, such threshold will diminish as populations depart from maximum numbers predicted by RF models (the departure is governed by the interaction term of the model). Finally, if population disruption is high, regression lines will have strong negative slopes even at low temperatures.

**Table S6.** Summary of the best general regression models explaining variations in the slope of the regression line between maximum temperature and residuals from RF models by life stages.

| **Response variable** | **Model summary** | **Predictors** | **Coefficients** | ***P*** |
| --- | --- | --- | --- | --- |
| Slope YOY | *R*^2^ = 0.46; *F* = 13.5; | Intercept | 138.6 | 0.24 ns |
|  | *d.f.* = 47; *P* < 0.001 | Max *T_max7d-water_* | -105.1 | 0.025 * |
|  |  | Mean Residuals | 396.5 | 0.034 * |
|  |  | Interaction term | -290.0 | 0.040 * |
| Slope Juvenile | *R*^2^ = 0.53; *F* = 17.4; | Intercept | -81.1 | 0.07 ns |
|  | *d.f.* = 47; *P* < 0.001 | Max *T_max7d-water_* | 62.1 | 0.049 * |
|  |  | Mean Residuals | 269.9 | 0.024 * |
|  |  | Interaction term | -195.0 | 0.030 * |
| Slope Adult | *R*^2^ = 0.57; *F* = 31.1; | Intercept | 42.4 | 0.17 ns |
|  | *d.f.* = 48; *P* < 0.001 | Max *T_max7d-water_* | -39.9 | 0.017 * |
|  |  | Mean Residuals | 182.5 | 0.015 * |
|  |  | Interaction term | -129.5 | 0.017 * |

**References in Appendix S2**

Pinheiro J, Bates D, DebRoy S, Sarkar D, and the R Development Core Team (2011) nlme: linear and nonlinear mixed effects models [online]. R package version 3.1-102. Available: <http://cran.r-project.org/web/packages/nlme/index.html>. Accessed 2013 Oct 23.

Zuur AF, Ieno EN, Walker NJ, Saveliev AA, Smith GM (2009) Mixed Effects Models and Extensions in Ecology with R. Dordrecht: Springer-Verlag.
